# *In vivo* Morphometry of Inner Plexiform Layer (IPL) Stratification in the Human Retina With Visible Light Optical Coherence Tomography

**DOI:** 10.3389/fncel.2021.655096

**Published:** 2021-04-29

**Authors:** Tingwei Zhang, Aaron M. Kho, Vivek J. Srinivasan

**Affiliations:** ^1^Department of Biomedical Engineering, University of California, Davis, Davis, CA, United States; ^2^Department of Ophthalmology and Vision Science, School of Medicine, University of California, Davis, Sacramento, CA, United States; ^3^Department of Ophthalmology, NYU Langone Health, New York, NY, United States; ^4^Department of Radiology, NYU Langone Health, New York, NY, United States; ^5^Tech4Health Institute, NYU Langone Health, New York, NY, United States

**Keywords:** retina, inner plexiform layer, outer plexiform layer, retinal lamination, synapses, visible light optical coherence tomography, bipolar cells, ganglion cells

## Abstract

From the bipolar cells to higher brain visual centers, signals in the vertebrate visual system are transmitted along parallel on and off pathways. These two pathways are spatially segregated along the depth axis of the retina. Yet, to our knowledge, there is no way to directly assess this anatomical stratification *in vivo*. Here, employing ultrahigh resolution visible light Optical Coherence Tomography (OCT) imaging in humans, we report a stereotyped reflectivity pattern of the inner plexiform layer (IPL) that parallels IPL stratification. We characterize the topography of this reflectivity pattern non-invasively in a cohort of normal, young adult human subjects. This proposed correlate of IPL stratification is accessible through non-invasive ocular imaging in living humans. Topographic variations should be carefully considered when designing studies in development or diseases of the visual system.

## Introduction

The retina transmits and filters light-evoked signals from the two-dimensional photoreceptor mosaic to the output ganglion cells that relay visual signals to the brain. The function of the retina naturally gives rise to retinal stratification (Wassle, 2004), or laminar organization of neural circuitry that processes visual signals. For example, synapses are segregated from cell somas along the depth axis of the retina, being organized into two major layers: the outer plexiform layer (OPL) and the inner plexiform layer (IPL). The OPL contains synapses between the rod and cone photoreceptors and bipolar cells, with lateral interactions provided by horizontal cells. The IPL contains synapses between bipolar cells, amacrine cells, and the output ganglion cells. Additionally, each synaptic layer is further stratified; the OPL is divided into rod and cone synapses (Kolb, 1977), while the IPL is divided into ON (sublamina B) and OFF (sublamina A) bipolar cell axon terminations, which give rise to ON and OFF channels (Famiglietti and Kolb, 1976; Nelson et al., 1978) that nominally respond to light increments and decrements, respectively. The IPL is often further divided into 5 strata of approximately equal thickness, with the two innermost strata corresponding to the ON pathway (sublamina A), the two outermost strata corresponding to the OFF pathway (sublamina B), and the middle stratum designated as either ON or as a watershed zone (Balasubramanian and Gan, 2014). This pentalaminar scheme for describing the IPL, initially based on Müller glia transverse processes (Cajal, 1893; Polyak, 1941), has now become a *de facto* convention. In the primate retina, bipolar cell ramifications (Mariani, 1984; Boycott and Wässle, 1991; Kolb et al., 1992), assessed by Golgi staining, and neurotransmitters (Marc, 1986), assessed by autoradiography and immunostaining, support a pentalaminar organization. This scheme is also paralleled by synapse density (Koontz and Hendrickson, 1987), and dendritic tree distributions of cell types such as midget ganglion cells (Dacey, 1993). IPL lamination is often delineated *ex vivo* by immunostaining of various cell types (Weltzien et al., 2015); however, data on human IPL lamination are sparse (Haverkamp et al., 2003).

**FIGURE 1:**
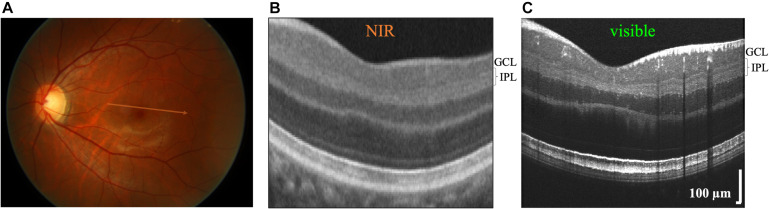
Commercial near-infrared (NIR) and visible Optical Coherence Tomography (OCT) of a 26 years old Asian male with brown-colored iris. **(A)** OCT generates cross-sectional images by scanning broad bandwidth light on the fundus of the retina. Commercial NIR OCT **(B)** and visible light OCT **(C)** images of similar retinal eccentricities, intersecting the foveal pit superior to the foveola. Compared to commercial NIR OCT, visible light OCT achieves fivefold finer axial resolution, which improves visualization of substrata within the inner plexiform layer (IPL). In the visible light OCT image, IPL stratification is evident everywhere except near the central foveal pit. The NIR OCT image **(B)** was cropped from a Zeiss Cirrus High Definition 5 Line Raster scan (approximate location shown on fundus image in **A**).

While the function of the ON and OFF pathways can be individually assessed non-invasively by electroretinography or electroencephalography (Norcia et al., 2020), there is no known *in vivo* methodology that can assess their anatomy. Perhaps the closest approach is Optical Coherence Tomography (OCT), a standard clinical imaging modality for *in vivo* high-resolution cross-sectional imaging of the human retina (Drexler and Fujimoto, 2008). Essentially, OCT scans a near-infrared (NIR) light beam on the retina to form images of backscattering or backreflection as a function of retinal depth and eccentricity (Figure 1A). Conveniently, the laminar organization of the retina, with synaptic layers alternating with nuclear layers, leads to differences in reflectivity (backscattering) that form the basis for OCT image contrast (Huang et al., 1991; Figure 1B). However, while the IPL and OPL are well-visualized in OCT, the internal structure of these layers has received little attention, aside from a few scattered reports noting the presence of IPL stratification (Tanna et al., 2010; Zhang et al., 2019; Miller and Kurokawa, 2020). One possible reason is that the changes in reflectivity that accompany stratification of synaptic layers are subtler than those that give rise to contrast between nuclear and synaptic layers (Tanna et al., 2010; Zhang et al., 2019; Miller and Kurokawa, 2020). Also, retinal stratification occurs on the micron scale, requiring depth resolution beyond the capabilities of most commercial NIR OCT systems to distinguish.

In this study, to investigate stratification of the IPL in normal human subjects, we employ a prototype ultrahigh resolution visible light OCT system (Zhang et al., 2019, 2020) with 1.0 micron axial resolution, finer than commercial near-infrared (NIR) OCT (5–7 micron resolution), and ultrahigh resolution NIR OCT prototypes (Lee et al., 2020) (2.7 micron resolution). We target this initial study to the central 7.5° of the human macula, which is critical for visual function, and where about 30% of all retinal ganglion cells are found (Curcio and Allen, 1990). Based on imaging the IPL, we report strata thicknesses and reflectivity patterns topographically in a cohort of human subjects without history or evidence of ocular pathology. Admittedly, a simple reflectivity pattern probably does not capture the rich complexity of the IPL. However, this correlate of functional stratification can be measured noninvasively, in the central nervous systems of living human subjects.

## Materials and Equipment

A prototype visible light OCT system (Zhang et al., 2019), developed at UC Davis, was employed for these studies. We incorporated software axial tracking, using the interference fringes for rapid calculation, to ensure that the retina is stabilized in an axial range for near-optimal sensitivity during imaging (Zhang et al., 2019). We implemented rapid spectral shaping using a Grating Light Valve Spatial Light Modulator (GLV-SLM) device (Zhang et al., 2019). The GLV-SLM helps to reduce the short wavelength light exposure of visible light OCT. With a rapid 108 kHz amplitude modulation, the GLV-SLM initially adjusts the source spectrum to fall between 600 and 650 nm (red-orange light) for subject alignment, in order to reduce rhodopsin bleaching, improve patient comfort, and reduce photochemical effects. Once aligned, a wider, 150 nm full spectral width (100 nm full-width-at-half-maximum) is employed for image acquisition with optimal axial resolution. Compared to the alternative approach of using an additional wavelength-multiplexed NIR OCT system for alignment (Song et al., 2018), the GLV-SLM approach is simpler and less expensive.

Informed consent was obtained from twenty adult human subjects without any history of ocular pathology. If both eyes of a single subject were imaged, only one eye was randomly chosen for inclusion in the analysis. Eyes were excluded from the study if the IPL itself was not uniformly visualized across the entire field-of-view of all six radial high quality images, indicative of poor SNR. Based on this criterion, four subjects were excluded. This exclusion rate is higher than prior NIR OCT studies that targeted the IPL and GCL (Woertz et al., 2020). Thus, a total of 16 eyes of 16 subjects were included in this study. The mean subject age was 27.7 ± 4.8 years (range from 23 to 40 years old), with 9 females and 7 males. This young adult cohort could serve as a baseline for future studies of development or aging. A pentalaminar pattern was discernable in all 16 eyes where the IPL was visualized. This research was approved by the UC Davis Institutional Review Board and conformed to the principles of the Declaration of Helsinki.

The OCT axial resolution is nominally determined by the coherence length of the light source. The OCT axial full-width-at-half-maximum (FWHM) resolution in air is given by δz_*air*_=0.44λ_0_^2^/dλ where λ_0_ is the central wavelength and dλ is the FWHM bandwidth. The axial resolution in tissue is given by δz_*tissue*_ =δz_*air*_/n_*tissue*_, where n_*tissue*_ is the group refractive index in tissue. For a fixed dλ, finer axial resolutions are enabled by a shorter wavelengths (Povazay et al., 2002). For instance, our FWHM bandwidth of 100 nm yields an axial resolution in air of 1.4 microns if centered at 565 nm, but just 3.2 microns if centered at 850 nm. Accounting for the tissue refractive index, our system achieves an axial resolution in tissue of 1.0 microns (*n*_*tissue*_=1.35), sufficient to examine stratification of the IPL. In a prior study, an NIR OCT axial resolution of 1.4 microns in tissue (*n*_*tissue*_=1.33) was reported by Tanna et al. (2010) although their measurements of external limiting membrane (ELM) thickness suggested that a slightly coarser resolution was realized *in vivo*. Based on the data provided (Tanna et al., 2010), and assuming an infinitesimally thin intrinsic ELM, we estimate that this prior study achieved an effective axial image resolution of 2.4 microns in tissue.

In addition to the nominal OCT axial resolution, axial resolution changes with imaging depth must also be considered (Lee et al., 2020). Using a novel method of calculating spectral resolution from excess noise correlations of a supercontinuum light source in real time (Kho et al., 2020), employing simple off-the-shelf achromats for the focusing lens of the spectrometer, we achieved a flat spectral resolution at all wavelengths in the visible OCT spectrum, leading to a uniform axial resolution across depth (Zhang et al., 2020). With this improved alignment, the sensitivity drop was ∼3.4 dB/mm in air and the axial resolution degradation was about 5% over the first millimeter in air. Practically, this means that OCT depth resolution does not change appreciably with eye motion, aiding reproducibility and accuracy of our morphometric measurements. Together with axial tracking (discussed above), and water wavenumber calibration (discussed below), the improved spectrometer alignment ensured that ultrahigh image resolution was realized *in vivo* by our visible light OCT system.

## Materials and Methods

### Scanning Protocol

Visualization of IPL lamination in OCT presents very specific and unique challenges. Depending on subject and retinal eccentricity, the intensity contrast of the hyporeflective bands can range from 5 to 40% with respect to the hyperreflective bands in the IPL. Major sources of noise include speckle and additive noise. Speckle arises from the random interference of unresolved light fields backscattered from the same coherence volume, whereas additive noise arises from the light source and/or the sensor. The contrast (standard deviation divided by the mean) of fully developed speckle is 100%. Therefore, a scanning protocol must achieve spatial diversity, to reduce speckle and improve the visualization of the IPL. To achieve this, we opted to acquire a raster scan, with a series of fast frames separated along the slow axis perpendicular to the fast scan direction, which are then motion-corrected and intensity-averaged to form a single high quality image, wherein IPL lamination was quantified. Important variables to consider include the imaging speed, the total number of frames, and the frame spacing. An imaging speed of 30 kHz was chosen to reduce motion, while providing sufficient signal to distinguish the subtle IPL strata in the presence of additive noise. A total of 30 frames were acquired to ensure a reasonable image acquisition time. The frame spacing was 5.2 microns along the perpendicular (slow) axis, corresponding to a total slow axis eccentricity range of approximately 0.5°. This frame spacing ensured that the frames were minimally correlated, helping to reduce speckle after motion correction and intensity averaging. In order to optimize axial image resolution, OCT images were reconstructed using water wavenumber calibration and transverse dependent dispersion compensation (Zhang et al., 2020).

To acquire topographical information, we acquired six raster scans, as described in the previous paragraph, angled at intervals of 30° in a radial spoke pattern, across the macula. The center of the spoke pattern was aimed at the foveola. As the scan pattern did not always intersect the foveola due to fixation error, we performed two additional analyses. In the first analysis, we performed a global correction of eccentricities, defining the foveolar center as the position of minimal distance between the inner limiting membrane (ILM) and the inner segment/outer segment junction (IS/OS) as previously described (Zhang et al., 2021). Second we analyzed lamination patterns according to IPL thickness. IPL thickness vanishes in the foveal center, precipitously increases along the foveal slope, exhibits a broad maximum around 1–2 mm eccentricity, and gradually decreases more eccentrically (Curcio et al., 2011; Moura et al., 2012). Therefore, while IPL thickness and eccentricity are related, IPL thickness is not a direct proxy for eccentricity as there is no monotonic relationship between the two. The analysis of IPL lamination according to IPL thickness was viewed as being more robust than the eccentricity-based analysis since thickness measurements were co-registered with laminar profiles.

As illustrated in Figure 1, visualizing IPL lamination in the fovea is particularly challenging. This difficulty is due in part to limited axial resolution. Also, anatomy changes rapidly near the foveal pit. To average a sufficient number of speckles to reduce speckle noise to acceptable levels, important anatomical details such as IPL lamination are blurred. Thus, while IPL lamination in the foveal pit might indeed exist, we were not able to report on it. Structure-function correlations will need to be planned with this limitation in mind in the future.

### Image Analysis

**FIGURE 2:**
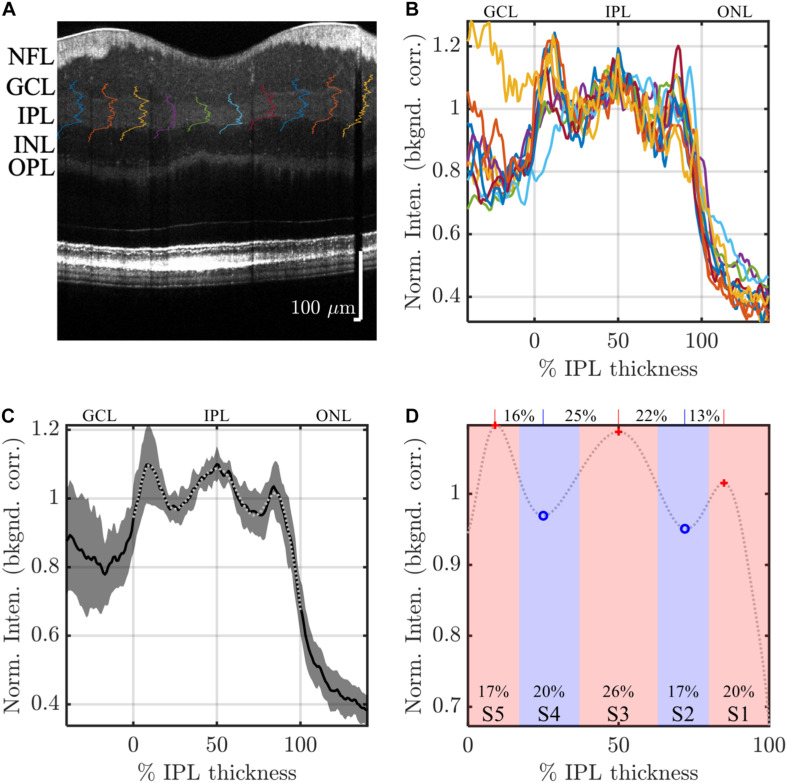
Image analysis: example analysis of IPL strata (S1-S5) in a high quality visible light OCT image. IPL intensity profiles (background corrected, averaged transversally over 1.5°, and normalized so the average IPL intensity is 1. are displayed across the image **(A)** and also plotted versus % IPL thickness **(B)**. **(C)** Average IPL profile, excluding segments with an IPL thickness below 24 microns, across locations (mean ± SD) shows a stereotyped pattern with 3 peaks and 2 valleys. A polynomial fit approximates the average profile (light gray dotted line), providing estimates of both peak and valley (extrema) locations **(D)**. In this example, the reflectivity peak at the center of S3 is broad, separated by 25 and 22% IPL thickness from the nearest inner and outer valleys, respectively, as shown on top of the plot. This broad peak is flanked by relatively narrower peaks at the centers of S1 and S5, which are separated from the nearest inner and outer valleys by only 13 and 16% IPL thickness, respectively. In agreement with this observation, a wider S3 was also noted, where stratum divisions were defined by positions where intensity crossed the midpoint between adjacent extrema (red and blue shading), as shown at the bottom of the plot.

The inner retinal layer boundaries were first delineated using a variant of a previously-described algorithm (Srinivasan et al., 2008), where the layer edges were defined by zero crossings of the second derivative of the OCT image intensity. Errors were corrected manually (Moura et al., 2012; Woertz et al., 2020). First, OCT intensity in the high quality image was background-corrected to remove the bias caused by additive noise. Next, to enable consistent comparisons of stratification across varying IPL thicknesses, at each transverse position, the IPL intensity was linearly interpolated onto a thickness percentage abscissa axis, with 0% representing the IPL-ganglion cell layer (GCL) boundary and 100% representing the IPL-inner nuclear layer (INL) boundary. Note that this IPL thickness percentage axis has 1% increments. Thus, even for a thick IPL of 50 microns, a fine sampling interval of 0.5 microns after linear interpolation was ensured. Note that percentages less than 0% and greater than 100% corresponded to the GCL and the INL, respectively. Images were segmented into transverse regions of 450 microns (1.5°) and IPL intensities were averaged on the IPL percentage thickness axis, across each segment. To ensure consistent weighting of segments, each segment was normalized to achieve a mean IPL intensity of 1 in segmental intensity profiles (Figures 2A,B). With this normalization, IPL intensities could be interpreted as contrasts relative to the mean IPL intensity. After this series of steps, five IPL layers were consistently observed in segmental IPL intensity profiles (Figures 2A,B), whenever the mean segment IPL thickness was greater than 24 microns.

To further reduce noise and detect salient features of the intensity profiles, a 14th order polynomial fit was performed on the mean segmental intensity profile (101 points from 0 to 100%) within the IPL (gray dotted line in Figure 2C). This fit faithfully represented the pentalaminar intensity pattern of the IPL, with three local maxima (peaks) and two local minima (valleys), and removed some of the extraneous fluctuations in the profile related to speckle or additive noise (Figure 2C). The R^2^ of this fit was correlated with the image signal-to-additive noise ratio, supporting that the residual, unexplained variance removed by the fit was at least partially related to noise (data not shown). The polynomial fit provided ready access to features such as stratum location (the locations of local extrema) and stratum contrast (the ratios of local extrema), facilitating comparisons across locations and subjects. As described next, stratum location was further analyzed to determine thicknesses of S1–S5.

Though IPL strata are often assumed to be approximately equal, we sought to empirically investigate stratum thicknesses based on the IPL reflectivity profile. As there is no clear *a priori* definition of stratum thickness, we chose to investigate two reasonable, but slightly different, approaches to assess thickness. In the first approach, stratum boundaries were defined as the positions where the intensity profile crossed the midpoint between adjacent peaks and valleys (Figure 2D, red and blue shading). Given a total of 4 boundaries between 5 local extrema, an inner boundary at 0%, and an outer boundary at 100%, this approach yielded 6 boundaries that delineated the 5 IPL strata (S1–S5) on the basis of reflectivity. In the second approach, the distances between adjacent extrema (Figure 2D, red crosses and blue circles) were determined, leading to 4 thickness values for transitions (S1–S2, S2–S3, S3–S4, and S4–S5), as shown across the top of Figure 2D. Though the thickness values determined by the second approach corresponded to transitions between adjacent strata, not to individual strata *per se*, they provided a consistency check for the first approach.

**FIGURE 3:**
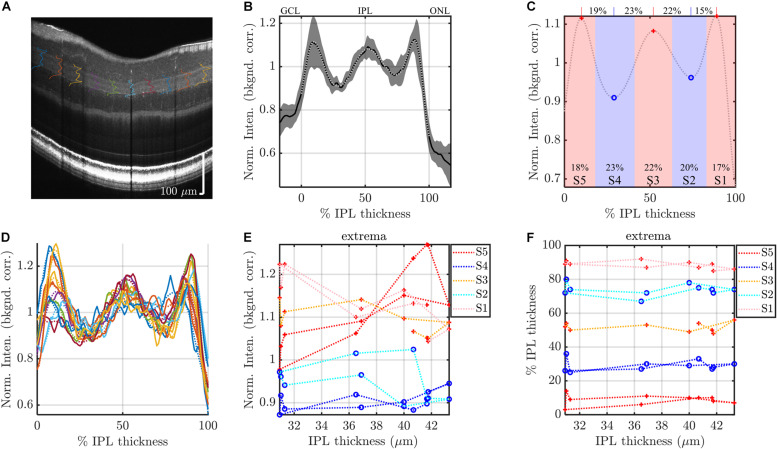
Segmental analysis: example analysis of IPL segments to evaluate topography. **(A)** IPL intensity profiles (background corrected, averaged transversally over 1.5° segments, and normalized) are displayed across the image. As in Figure 2, the average IPL profile **(B)**, with polynomial fit and derived stratification parameters **(C)**, are shown. **(D)** To evaluate topography, individual 1.5° segments are analyzed. Each segmental polynomial fit (dotted lines in **D**) provides extrema **(E)** and their locations **(F)**, using the image-averaged profile **(C)** as a template (see main text). Red crosses are segmental maxima and blue circles are segmental minima. This segmental analysis reveals variations in stratification with either IPL thickness (abscissa in **E,F**) or eccentricity. Data from consecutive segments are connected with dotted lines.

With five extrema, five stratum thicknesses, and four inter-stratum transitions, we evaluated a total of 14 parameters to characterize the internal IPL reflectivity on visible light OCT. The extraction of these parameters, which involves fitting and peak detection as described above, is sensitive to noise. Therefore, we analyzed the IPL intensity profile with varying degrees of averaging: (1) we analyzed intensity profiles, averaged across all subjects by eccentricity or IPL thickness (most averaging) to extract parameters, (2) we analyzed intensity profiles, averaged across each high quality image (intermediate averaging, 96 total images) to extract parameters, or (3) we analyzed raw segmental intensity profiles (least averaging, 960 total segments) to extract parameters (Figure 3). This first and second analyses were less susceptible to noise because of the increased averaging. Note that all averaging was performed on 1.5° segments, after normalization as described above, to ensure equal weighting of the segments. However, because the IPL patterns did not align exactly across different subjects and eccentricities, the first and second analyses resulted in a slight loss of contrast relative to the third. Findings were viewed as being robust if they were supported by all analysis methods.

Data were analyzed in Matlab (Natick MA). To determine extrema from the IPL profiles, we used the findpeaks function on the 14th order polynomial fitted IPL profiles, with the additional constraint that the distance between consecutive maxima or consecutive minima must be greater than 25% of the IPL thickness. For three out of 96 average image profiles, the extrema did not match the template of peak-valley-peak-valley-peak. These images were discarded. For the individual analysis of the segmental intensity profiles (least averaging), shown in Figure 3, we used the average image profile (Figure 3C) as a “template” to guide analysis. The search for extrema for each segmental profile (Figure 3D) was constrained to fall within the previous and next extrema of the average profile and within the confines of the IPL. For instance, the S2 minimum searches for the segmental profiles were constrained to fall between S1 and S3 maxima of the average profile in the image. Likewise, the S5 maximum searches were constrained to fall between the S4 minimum and the inner boundary of the IPL. Extrema at the edge of the search range were considered to be invalid and discarded. As above, extrema in segmental profiles which deviated from the template of peak-valley-peak-valley-peak were discarded. We also discarded all 30 segments (3 images × 10 segments) where the average image profile template did not show a clear pentalaminar pattern. Taken together with the constraint that IPL thickness exceeded 24 microns, these criteria resulted in discarding about 17% of the stratum extrema, and about 26% of the stratum transitions. Exclusion of data was deemed necessary due to the noise in segmental IPL profiles (as exemplified in Figure 3D). Once again, to alleviate the concern that discarding data might introduce biases, data were also analyzed with more averaging, as described above.

### Statistics

To assess differences between strata, parameters were compared using analysis of variance (ANOVA) with Tukey’s Honest Significant Difference test. To rigorously model subject differences, we used linear models both with (mixed) and without (fixed) random effects. For these models, segments with an IPL thickness of less than 24 microns were excluded. In addition to the criteria for outlier exclusion discussed in Image Analysis, we also excluded data points where the fitting residual was greater than 5 standard deviations of the residual fit. All models were checked for homoscedasticity of residuals and valid confidence intervals for all estimated parameters. Aikake’s information criterion was used when comparing competing models.

## Results

**FIGURE 4:**
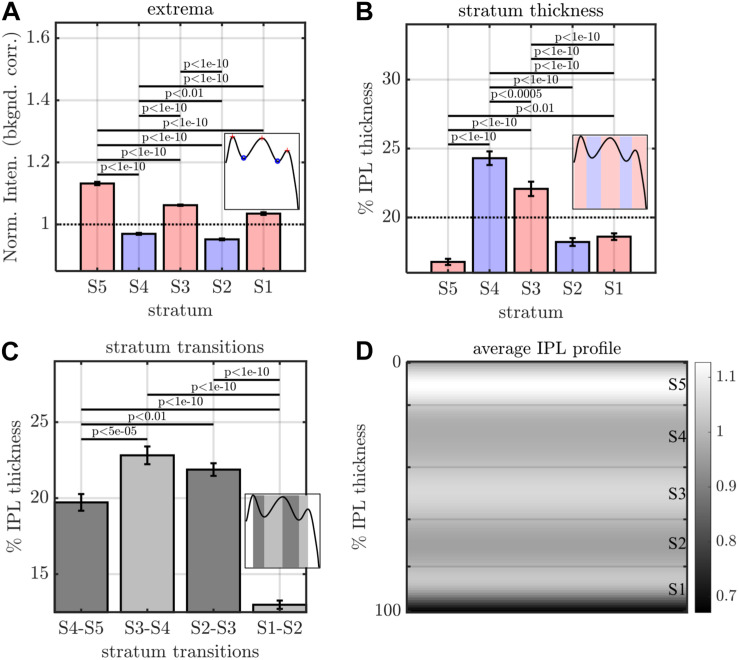
Image analysis of IPL lamination parameters across 96 images (16 eyes × 6 high quality radial images), derived from the averaging and fitting procedure shown in Figure 2. **(A)** A stereotyped reflectivity pattern is consistently observed. Stratum thickness **(B)** and inter-stratum transitions **(C)** suggest a broadening around S3–S4. **(D)** Average image profile (shown as intensity image) clearly depicts the major features (i.e. high intensity or prominent S5, broad S3 and S4, narrow S1 and S5. Horizontal lines denote stratum boundaries.

**FIGURE 5:**
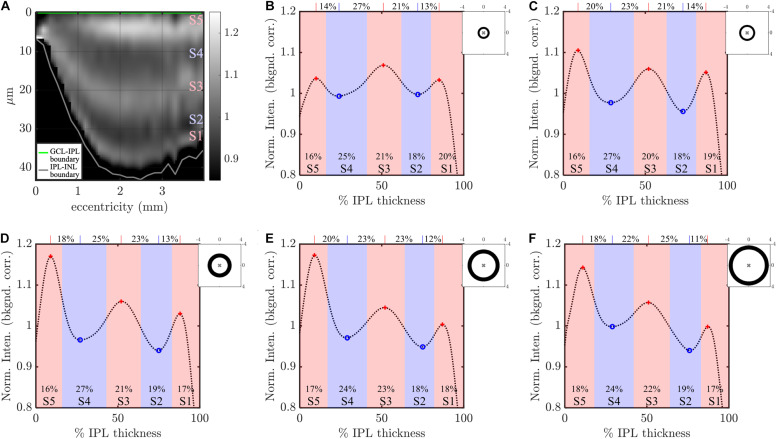
Eccentricity-wise averaging of segmental IPL profiles. **(A)** Subject-averaged IPL lamination image, obtained by partitioning IPL segments into 25 eccentricity bins, averaging IPL profiles and thicknesses within each bin, then for each eccentricity bin, rescaling the abscissa of the average segmental profile to the average IPL thickness. IPL profiles, averaged across wider eccentricity bins (**B:** 0.75–1.13 mm, **C:** 1.13–1.5 mm, **D:** 1.5–2.25 mm, **E:** 2.25–3 mm, **F:** 3–3.75 mm). Both the image **(A)** and the plotted profiles **(B–F)** suggest an increase in the prominence of S5 starting near the foveal edge (0.75 mm). Note that while averaging across subjects and within eccentricity bins yields smooth profiles, individual profiles may not align; therefore, stratum contrast is reduced in this figure relative to Figures 6A, 7A. Topographic images in **(B–F)** show annuli for eccentricity binning relative to the foveal center (“x”).

**FIGURE 6:**
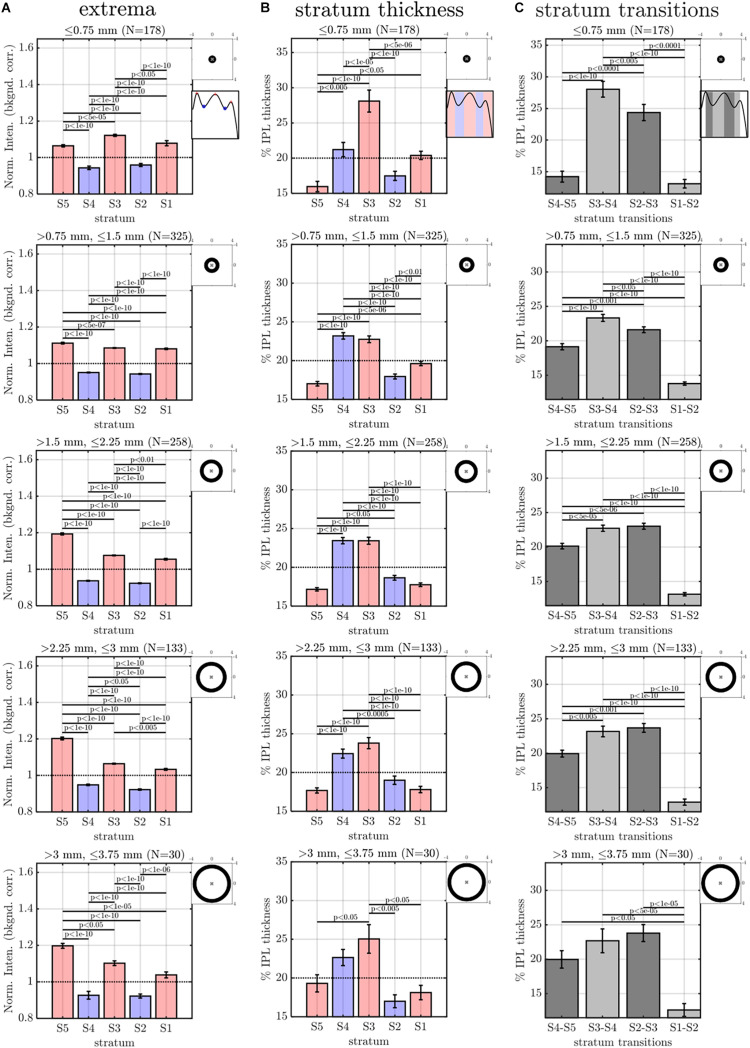
Eccentricity-wise summary of segmental IPL parameters, derived from the averaging and fitting procedure shown in Figure 3: extrema **(A)**, thicknesses **(B)**, and transitions between strata **(C)**. Note that since segmental profiles are not averaged before determining extrema, stratum contrast is increased relative to Figure 5, though the trends remain consistent. Topographic images show annuli for eccentricity binning relative to the foveal center (“x”).

**FIGURE 7:**
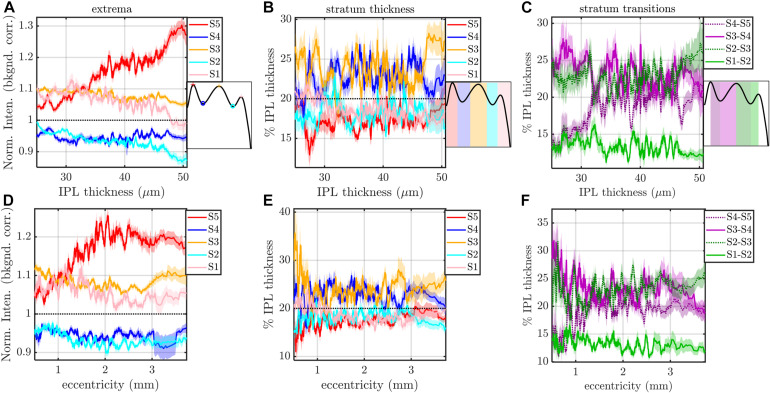
Rolling average (mean ± std. err., window size of 21) of IPL stratification parameters (extrema, thicknesses, and transitions) versus IPL thickness **(A–C)** and eccentricity **(D–F)**. The most salient feature is an increase in the intensity of S5 with IPL thickness **(A)** and an increase and pleateau in the intensity of S5 with eccentricity **(D)**. S3 and S4 are consistently thicker, regardless of IPL thickness **(B)** and eccentricity **(E)**. In agreement with these findings, S2–S3 and S3–S4 transitions are broader than other inter-stratum transitions **(C,F)**. An increase in the width of the S4–S5 transition **(C,F)** accompanies the increased extrafoveal prominence of S5 **(A,D)**.

**FIGURE 8:**
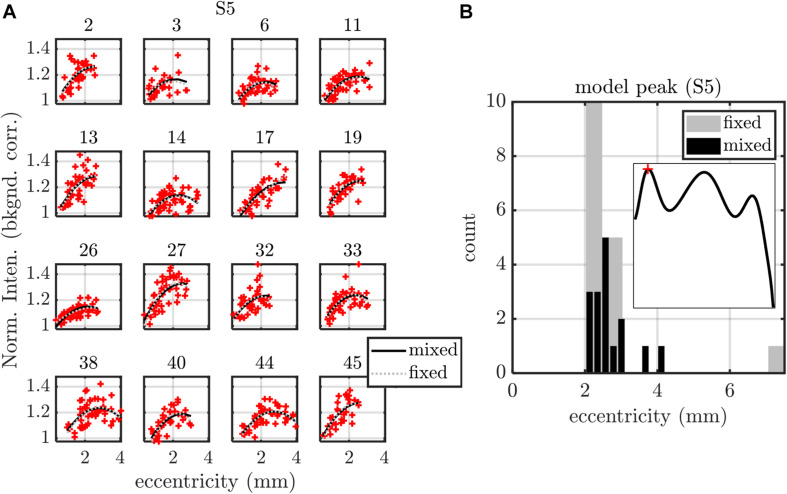
Summary of fixed (fixed intercept, slope, and quadratic term for each subject) and mixed (fixed intercept, slope, and quadratic term as well as random intercept, slope, and quadratic term for each subject) effects models, applied to S5 peak versus eccentricity. **(A)** Model fits are shown for each of the 16 subjects, with subject index in subplot titles. **(B)** Both fixed and mixed effects models predict a maximum of the parabolic S5 peak profile around 2.4 mm eccentricity. Models for all other stratification parameters are summarized in Table 1 (eccentricity as a predictor) and Table 2 (IPL thickness as a predictor).

**FIGURE 9:**
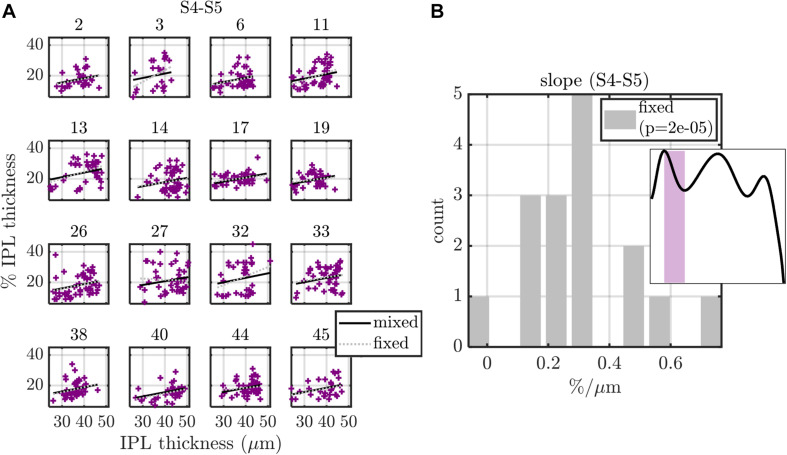
Summary of fixed (fixed intercept and slope for each subject) and mixed (fixed intercept and slope, with independent random intercept and slope for each subject) effects models, applied to S4-S5 transition width versus IPL thickness. **(A)** Model fits are shown for each of the 16 subjects, with subject index in subplot titles. **(B)** Histogram of subject slopes from the fixed effects model shows a statistically significant positive slope. Similar analysis for other stratification parameters are summarized in Table 1 (eccentricity as a predictor) and Table 2 (IPL thickness as a predictor).

We present our results in order of increasing complexity, starting first with IPL profile parameters determined on an image-by-image basis (Figure 4), the average IPL profile by eccentricity (Figure 5), IPL profile parameters by eccentricity (Figures 6, 7) and IPL thickness (Figure 7), and finally, subject-specific modeling of IPL parameters (Figures 8, 9 and Tables 1, 2).

**TABLE 1:** Summary of slopes versus eccentricity and their *p*-values for both fixed (fixed intercept and slope for each subject) and mixed (fixed intercept and slope, with independent random intercept and slope for subject grouping) effects models.

**Model Type**	**Slopes vs. eccentricity**	**Extrema (normalized intensity)**					**Stratum thickness (% IPL thickness)**					**Transition (% IPL thickness)**			
		**S5**	**S4**	**S3**	**S2**	**S1**	**S5**	**S4**	**S3**	**S2**	**S1**	**S4–S5**	**S3–S4**	**S2–S3**	**S1–S2**
Fixed effects	vs. eccentricity (mm)	N/A	0.0040	−0.016	−0.022	−0.037	0.57	−0.30	0.33	0.95	−1.2	2.2	−1.9	2.0	−0.92
	*p*-value	N/A	0.3	0.0001	0.0002	1E-05	0.2	0.5	0.6	0.02	0.002	0.001	0.01	0.001	0.03
Mixed effects	vs. eccentricity (mm)	N/A	0.0041	−0.015	−0.021	−0.036	0.60	−0.51	0.61	0.62	−1.08	1.8	−1.5	1.8	−0.85
	*p*-value	N/A	0.2	1E-06	9E-07	6E-13	0.1	0.2	0.2	0.1	0.0001	0.0002	0.002	0.0001	0.01

*Note that the S5 peak was analyzed separately in Figure 8 with quadratic models and is not included here. A positive slope indicates an increase in the parameter with eccentricity. Both models indicate that the S1–S3 extrema decrease with eccentricity, that the thickness of S1 decreases with eccentricity, and that the S4–S5 and S2–S3 transition widths increase with eccentricity, while the S1–S2 and S3–S4 transition widths decrease with eccentricity. The slope unit is the column unit divided by the row unit (mm).*

**TABLE 2:** Summary of slopes versus IPL thickness and their *p*-values for both fixed (fixed intercept and slope for each subject) and mixed (fixed intercept and slope, with independent random intercept and slope for subject grouping) effects models.

**Model Type**	**Slopes vs. IPL thickness**	**Extrema (normalized intensity)**					**Stratum thickness (% IPL thickness)**					**Transition (% IPL thickness)**			
		**S5**	**S4**	**S3**	**S2**	**S1**	**S5**	**S4**	**S3**	**S2**	**S1**	**S4–S5**	**S3–S4**	**S2–S3**	**S1–S2**
Fixed effects	vs. thickness (μm)	0.010	5.9E-05	–1.8E-03	–3.3E-03	–4.0E-03	0.064	0.056	0.060	−0.002	−0.15	0.31	−0.16	0.094	−0.12
	*p*-value	3E-10	0.8	0.0001	2E-07	6E-08	0.2	0.3	0.3	1.0	0.001	0.00002	0.03	0.1	0.01
Mixed effects	vs. thickness (μm)	0.01	2.2E-04	–1.8E-03	-3.3E-03	–4.0E-03	0.045	0.060	0.026	0.018	−0.15	0.28	−0.17	0.094	−0.10
	*p*-value	7E-43	0.3	3E-08	3E-20	9E-23	0.3	0.2	0.6	0.6	1.8E-06	6E-10	0.002	0.04	0.003

*A positive slope indicates an increase in the parameter with IPL thickness. Both models indicate that the S5 peak increases, while S1–S3 extrema decrease with IPL thickness, and that the thickness of S1 decreases with IPL thickness, and that the S4–S5 transition width increases with IPL thickness, while the S1–S2 and S3–S4 transition widths decrease with IPL thickness. The slope unit is the column unit divided by the row unit (μm).*

The image-averaged IPL profile analysis showed a characteristic pentalaminar pattern (Figure 4A), with three hyper-reflective strata (S1, S3, and S5) separated by two hypo-reflective strata (S2 and S4). S3 and S4 were thicker than the other strata (Figure 4B), while the S1-S2 transition was thinner than the other transitions (Figure 4C). All of these features were readily visible on the average IPL profile, displayed as a linear scale image (Figure 4D). Next, to investigate topography, we displayed the eccentricity-wise average IPL profile as a linear scale image (Figure 5A). While the averaging within each eccentricity bin was performed on a percent IPL thickness scale, for image display, the final average profile for each eccentricity bin was rescaled to the average IPL thickness for the corresponding eccentricity bin. Thus, Figure 5A shows the “average” appearance of the IPL in OCT, both in terms of thickness and stratum contrast. The average IPL profiles, determined with larger eccentricity bins (Figures 5B–F), reinforce the major trends seen in the image: an increase in the contrast of S5 with eccentricity starting at the foveal edge and plateauing in the perifovea, a consistently thicker S3 and S4, and a thinner S5. We next extended the analysis to determine parameters for each segment. Though the segmental IPL profiles were intrinsically noisier and some had to be excluded (see section “Materials and Methods”), this approach enabled statistical comparisons of IPL parameters at different eccentricities (Figure 6). Overall, the statistical comparisons confirmed the major qualitative observations from Figure 5; namely that S5 prominence increases at the foveal edge (Figure 6A), and that S3 and S4 are relatively thicker while S5 is thinner (Figure 6B). In addition, S2–S3 and S3–S4 transitions are shown to be relatively thicker while S1–S2 is thinner (Figure 6C). Rolling average plots recapitulated these trends, whether the abscissa was IPL thickness (Figures 7A–C) or eccentricity (Figures 7D–F). The rolling average plots also clarified that the prominence of S5 starts increasing at 0.75 mm eccentricity, reaching a broad plateau around 2–3 mm eccentricity with a possible decrease thereafter (Figure 7D). A concomitant increase in the S4–S5 transition width, around 1 mm eccentricity, was also noted (Figure 7F).

Finally, we applied mixed and fixed effects models to rigorously model subject-by-subject differences. Based on the results in Figure 7, we investigated both IPL thickness and eccentricity as independent variables or predictors. With a total of 14 parameters (dependent variables) to choose from and two predictors (independent variables) to choose from, we analyzed a total of 28 different data sets with different combinations of dependent and independent variables. With the additional option to model random effects or not, we created a total of 56 models.

We first noted that the S5 peak versus eccentricity data set required the inclusion of a quadratic term in the independent variable, whereas no other data sets did. This data set was treated as a special case, shown in Figure 8. The fixed effects model included a fixed intercept, slope, and quadratic term for each subject (3 × 16 = 48 parameters in total). The mixed effects model included a single fixed intercept, slope, and quadratic term, as well as a random intercept, slope, and quadratic term for each subject (51 parameters). For the mixed effects model, the random terms were assumed to be zero mean, normally distributed, and independent. The estimated coefficient of the quadratic term was always found to be negative for both models, predicting a local maximum (Figure 8A). Therefore, we determined the eccentricity where the modeled S5 contrast was maximized for each subject (Figure 8B). Both models yielded consistent results for the peak S5 eccentricity, typically ranging from 2 to 3 mm.

Excepting the one data set with S5 peak and eccentricity as the respective dependent and independent variables, all other 27 data sets were well fit by models with just an intercept and slope. For these data sets, we fit both a fixed effects model (16 fixed intercepts and 16 fixed slopes) as well as a mixed effects model (1 fixed intercept, 1 fixed slope, 16 random intercepts, and 16 random slopes). For the mixed effects model, the random intercept and slope were assumed to be zero mean, normally distributed, and independent. An exemplary analysis for S4–S5 transition thickness versus IPL thickness is shown in Figure 9. Predictions from both the fixed and mixed effects models are shown for each subject (Figure 9A). The histogram of fixed slopes is greater than 0, as revealed by a two-tailed *t*-test that the subject slopes were different than 0 (Figure 9B). A similar approach was used for all 27 data sets, as summarized in Tables 1, 2. For the fixed effects models, the mean value of the 16 subject slopes and corresponding *p*-value is shown. For the mixed effects models, the fixed slope estimate and its corresponding *p*-value is shown. Both models indicate that the S1–S3 extrema decrease with eccentricity/IPL thickness, though this decrease is much smaller in magnitude than the increase in the S5 peak with eccentricity (Figure 8) and IPL thickness (Table 2). Both models also indicate that the thickness of S1 decreases with eccentricity/IPL thickness, and that the S4–S5 transition width increases with eccentricity/IPL thickness, while the S1–S2 and S3–S4 transition widths decrease with eccentricity/IPL thickness. While statistically significant, these trends are nonetheless small.

## Discussion

This is the first systematic effort to comprehensively quantify the reflectivity pattern inside the IPL using OCT. Importantly, we find that IPL lamination was quantifiable in all eyes where the IPL could be visualized by our visible light OCT prototype. Therefore, IPL lamination is neither incidental nor anecdotal (Tanna et al., 2010; Zhang et al., 2019; Miller and Kurokawa, 2020), but rather, a common finding in ultrahigh resolution visible light OCT.

Since the cell subtypes in the IPL change as the retina transitions from a cone-dominated fovea to rod-dominated periphery, it is conceivable that the IPL reflectivity pattern may change as well. Indeed, we found that contrasts of the IPL strata changed with eccentricity and IPL thickness. The most salient trend was the increase in the contrast of S5 with eccentricity, starting from 1.05 at the edge of the fovea, and increasing to a plateau of 1.2 by 2.4 mm eccentricity, with a slight decrease thereafter. Given that rod bipolar cells stratify in the inner IPL (Boycott and Wässle, 1991; Kolb et al., 1992; Grunert et al., 1994), it is interesting to note that S5 is more prominent at eccentricities where the density of rod bipolar cells is higher (Boycott and Wässle, 1991; Kolb et al., 1992; Grunert et al., 1994). In interpreting these results, however, we must also keep in mind that the IPL profile was normalized, hence the prominence of S5 is only determined in reference to the other strata. Therefore an attenuation of other IPL strata with relatively more cone circuitry could also make S5 appear more prominent (Grunert et al., 1994).

**FIGURE 10:**
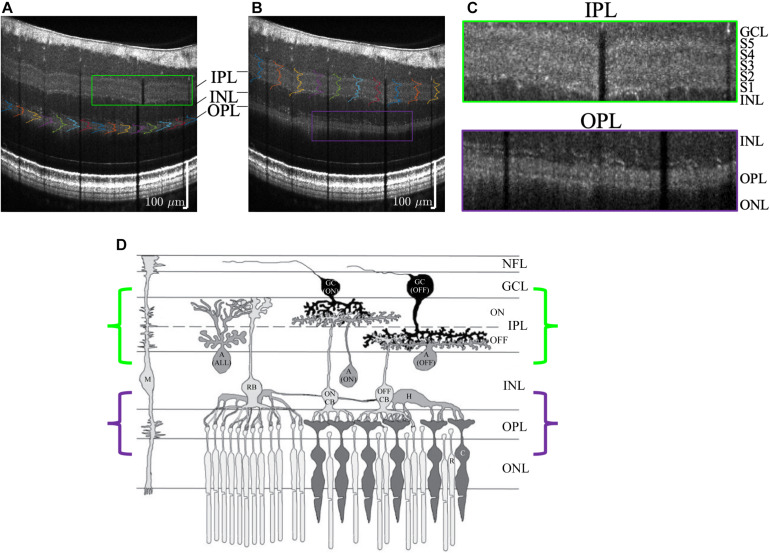
Visualization of outer plexiform layer (OPL) lamination **(A)** and inner plexiform layer (IPL) lamination **(B)** in the same high quality visible light OCT image. **(C)** Zooms show a pentalaminar IPL reflectivity pattern (green outline) and a trilaminar OPL reflectivity pattern (purple outline). **(D)** Anatomical diagram (reproduced with permission Wang et al., 2003) of retinal circuitry depicts ON-OFF IPL stratification and rod-cone OPL stratification, where the rod spherules are outer to the cone pedicles. Note that the diagram is drawn with bottom-up processing for consistency with the OCT image display (GC, ganglion cell; A, amacrine cell; M, Müller cell; H, horizontal cell; CB, cone bipolar cell; RB, rod bipolar cell; C, cone; R, rod).

The IPL is typically assumed to be partitioned into approximately equal strata (Polyak, 1941; Koontz and Hendrickson, 1987). This assumption implies that each stratum should occupy about 20% of the IPL thickness. Instead, we found that S3 and S4 occupy more of the IPL (21–25% each) at all eccentricities where lamination could be quantified. The broader S3 and S4 was also self-evident on individual OCT images (e.g., Figures 10A,C). Analysis of inter-stratum transitions led to a similar conclusion, with S3–S4 and S2–S3 consistently being the broadest transitions. The width of the S4–S5 transition increased with eccentricity immediately outside the fovea, coinciding with the increase in S5 contrast discussed above. Trends observed on a per-subject basis were also corroborated when averaging across subjects, bolstering confidence in our results.

Though the focus of this work was the IPL, we also found that visible light OCT often depicted lamination of the outer plexiform layer (OPL) too (Figures 10A–C). In the OPL, the rod spherules are reported as being organized in between and outer to the cone pedicles (Boycott and Wässle, 1991; Kolb, 1995), as is also suggested in Figure 10D (Wang et al., 2003). The mechanism for OPL stratification on visible light OCT requires further investigation.

Looking forward, we caution that the reflectivity correlate of IPL stratification arises from the optical properties, not functional properties, of strata in the IPL. Similarly, membrane and lipid stains show IPL strata (Marc, 1986), and differential interference contrast microscopy can also depict IPL sublamination (Gregg et al., 2013) *ex vivo*. We expect that laminar differences in synapse density size and morphology, or in neurite orientation, size, and density, as well as the refractive index of different neurites, may be responsible for the observed OCT reflectivity contrast *in vivo*. Mitochondria (Wong-Riley, 2010), Müller cells, and microvasculature could further modulate the observed reflectivity pattern (Though the intermediate capillary plexus is just outer to IPL strata S1; Campbell et al., 2017). Thus, the observed IPL reflectivity pattern likely arises from the aggregate of microstructural components that give rise to refractive index variations. On the negative side, it is probable that this reflectivity pattern has limited specificity for changes in subtypes of cells or to subtle changes in ramification patterns in the IPL. On positive side, however, the proposed method reveals IPL organization in living human subjects. Normal topographic variations should be considered when studying stratification during development and in diseases that affect the retina.

## Study Limitations

Given the restricted acquisition rate of visible light OCT in this study, we chose to target the macular region, which contains the highest density of ganglion cells in the retina, and is also a locus for glaucomatous damage (Hood et al., 2013). This study did not examine IPL lamination outside the macula, and this remains a topic for future investigation. Also, 20% of imaged subjects were excluded from the study due to low signal level that precluded detection of the IPL and its strata. More optimal scan protocols tailored to detect IPL stratification could help to improve yield in the future. Related to this issue, the more granular subject-wise analysis did require discarding more data; however, the major findings were bolstered by alternative analyses that did not discard data. Additionally, the IPL segmentation software in this study was not fully automated and required manual correction. A more automated segmentation software will enable more extensive studies in the future.

## Data Availability Statement

The raw data supporting the conclusions of this article will be made available by the authors, without undue reservation.

## Ethics Statement

The studies involving human participants were reviewed and approved by the UC Davis Institutional Review Board. The patients/participants provided their written informed consent to participate in this study.

## Author Contributions

VS and TZ designed the experiments and conducted the experiments. VS, TZ, and AK analyzed the images and data, wrote, and edited the manuscript. All authors contributed to the article and approved the submitted version.

## Conflict of Interest

VS receives royalties from Optovue, Inc. The remaining authors declare that the research was conducted in the absence of any commercial or financial relationships that could be construed as a potential conflict of interest.
